# Orbital angular momentum analysis for giant spin splitting in solids and nanostructures

**DOI:** 10.1038/s41598-017-02032-4

**Published:** 2017-05-17

**Authors:** Sehoon Oh, Hyoung Joon Choi

**Affiliations:** Department of Physics and IPAP, Yonsei University, Seoul, 03722 Korea

## Abstract

Giant spin splitting (GSS) of electronic bands, which is several orders of magnitude greater than the standard Rashba effect has been observed in various systems including noble-metal surfaces and thin films of transition-metal dichalcogenides. Previous studies reported that orbital angular momentum (OAM) is not quenched in some GSS materials and that the atomic spin-orbit interaction (SOI) generates spin splitting in some solid states via the interorbital hopping. Although the unquenched OAM may be closely related to the interorbital hopping, their relationship is hardly studied in the aspect of using the unquenched OAM as a control parameter of GSS. Here, we analyze OAM in GSS materials by using the interorbital-hopping mechanism and first-principles calculations. We report that the interatomic hopping between different-parity orbitals, which is generated by specific broken mirror symmetry, produces *k*-dependent OAM, resulting in valley-dependent GSS in WSe_2_ monolayer, Rashba-type GSS in Au (111) surface, and Dresselhaus-type GSS in bulk HgTe. We also demonstrate systematic control of OAM by pressure, external fields, and substrates, thereby controlling the spin splitting, and discuss the temperature dependence of OAM. Our results provide a simplified picture for systematic design and control of GSS materials.

## Introduction

Nonmagnetic crystals with inversion symmetry have electronic band structures with spin degeneracy^1^. When the inversion symmetry is broken, the spin-orbit interaction (SOI) can lift the degeneracy, generating an energy splitting^2–5^. In some materials, this splitting is several orders greater than that in conventional materials, which is referred to as the giant spin splitting (GSS)^6–23^. Since it is promising for applications such as room-temperature spintronic devices, the interest in the phenomenon has been increasing^24^.

According to the Rashba model^4, 5^, electrons moving in a plane perpendicular to an external electric field ${\overrightarrow{E}}_{ext}$ have momentum-dependent spin splitting of the form ${H}_{R}=\alpha ({k}_{x}{\sigma }_{y}-{k}_{y}{\sigma }_{x})$ originating from the SOI Hamiltonian ${H}_{SO}=\frac{e}{2{m}^{2}{c}^{2}}\overrightarrow{S}\cdot {\overrightarrow{E}}_{ext}\times \overrightarrow{p}$. Here *α* is a constant, *σ*_*x*_ and *σ*_*y*_ are Pauli matrices, and $\overrightarrow{k}=({k}_{x},{k}_{y})$, −*e*, *m*, $\overrightarrow{S}$, and $\overrightarrow{p}$ are the electron wavevector, charge, mass, spin, and momentum, respectively. This model has been used to describe spin-orbit-induced splitting in many materials^6, 9, 16, 20, 22, 25–27^. However, the splitting size from *H*_*SO*_ is too small to explain GSS if *E*_*ext*_ in *H*_*SO*_ is replaced with the symmetry-breaking part of internal and external electric fields. For example, the measured energy splitting in Au (111) surface states is about 10^5^ times what is expected from *H*_*SO*_ due to the surface electric field^6, 8, 10, 28, 29^. Furthermore, the spin splitting shows strong in-plane anisotropy in some materials, requiring sophisticated models^12, 22, 30^.

To generate GSS, atomic SOI due to strong electric field around nucleus is inevitable. Near a heavy element nucleus, the SOI Hamiltonian becomes ${H}_{SO}=\frac{e}{2{m}^{2}{c}^{2}}\overrightarrow{S}\cdot {\overrightarrow{E}}_{atom}\times \overrightarrow{p}=\lambda \overrightarrow{S}\cdot \overrightarrow{L}$, where ${\overrightarrow{E}}_{atom}$ is the electric field around the nucleus, *λ* is the atomic SOI strength, and $\overrightarrow{L}$ is the orbital angular momentum (OAM) of the electron near the nucleus. If an electronic state has a significant OAM near the heavy nucleus, GSS can take place. Previous studies on noble-metal surfaces and topological insulators reported that unquenched OAM coexists with GSS^28, 29, 31–33^, but not revealing the mechanism of the unquenching of the OAM clearly. Meanwhile, theoretical framework using interorbital hopping and atomic SOI^7, 34^ was extensively used for spin splitting in perovskite transition-metal oxide structures^35–38^. In these studies, unquenched OAM might originate straightforwardly from the interorbital hopping, but explicit study of unquenched OAM was not performed. In the study of ferroelectric halide perovskites using a similar framework^39^, significance of OAM was more recognized.

In this paper, we concentrate on the interorbital-hopping mechanism which produces OAM. We investigate the relation between the symmetry of the atomic structure and the unquenching of OAM and elaborate structural and orbital conditions for the emergence of unquenched OAM in solids and nanostructures. Once OAM is unquenched at atoms with large atomic SOI, it can produce GSS. We consider occurence of nonzero OAM in tight-binding analysis of atomic chains and analyze WSe_2_ monolayer, Au (111) surface, and bulk HgTe by first-principles calculations. Our results confirm the valley-dependent GSS in WSe_2_ monolayer, the Rashba-type GSS in Au (111) surface, and the Dresselhaus-type GSS in bulk HgTe. We also demonstrate that one can control OAM by modifying the atomic structure or lowering the symmetry by perturbations such as pressure, external electric fields, and substrates. Once OAM is modified, GSS is also modified. These results provide a simplified picture for systematic design and control of GSS materials.

## Results and Discussion

### Tight-binding model for unquenched OAM

**Figure 1:**
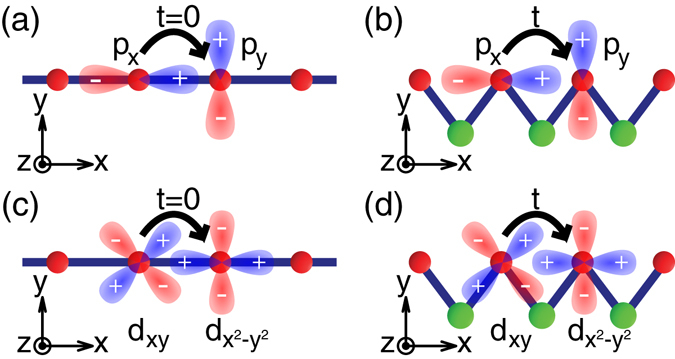
Interorbital-hopping mechanism for orbital angular momentum. (**a**,**b**) Atomic chains with two *p* orbitals at each red atom. (**c**,**d**) Atomic chains with two *d* orbitals at each red atom. In (**a**,**c**), the interatomic hopping between different-parity orbitals is zero due to a mirror plane parallel to the *xz* plane. In (**b**,**d**), the presence of green atoms does not remain any mirror plane parallel to the *xz* plane, producing nonzero interatomic hopping between different-parity orbitals.

As a heuristic example, we consider an atomic chain where each atomic site has *p*_*x*_ and *p*_*y*_ orbitals [Fig. 1(a)]. When the chain has a mirror plane parallel to the *xz* plane [Fig. 1(a)], the *p*_*x*_ and *p*_*y*_ orbitals are decoupled from each other and form two bands with zero OAM. In contrast, if the mirror symmetry is broken by the presence of other atoms [Fig. 1(b)], the *p*_*x*_ and *p*_*y*_ orbitals are coupled with each other, and the Hamiltonian, before considering SOI, can be expressed as

1 $${H}_{0}(k)=(\begin{matrix}{\varepsilon }_{{p}_{x}}+2{t}_{{p}_{x}{p}_{x}}\,\cos (ka) & 2i{t}_{{p}_{x}{p}_{y}}\,\sin (ka)\\ -\,2i{t}_{{p}_{x}{p}_{y}}\,\sin (ka) & {\varepsilon }_{{p}_{y}}+2{t}_{{p}_{y}{p}_{y}}\,\cos (ka)\end{matrix}),$$

where *k* is the wave number, *a* the lattice constant, *ε*_*p*_ the onsite energy of the *p*_*x*_ or *p*_*y*_ orbital, and *t*_*pp*′_ the nearest-neighbor hopping energy between *p* and *p*′ orbitals. Here, ${t}_{{p}_{x}{p}_{y}}$ is nonzero because of no mirror plane parallel to the *xz* plane. In addition, the *p*_*x*_ and *p*_*y*_ orbitals have different parity with respect to a plane normal to the chain, so the hopping energy from *p*_*x*_ to *p*_*y*_ in the −*x* direction has opposite sign to that in the +*x* direction. This results in *i* sin(*ka*) in the off-diagonal elements.

It is straightforward to diagonalize the Hamiltonian (1) to obtain band energies *E*_*n*_(*k*) and corresponding wavefunctions *ψ*_*n*_(*k*) and calculate the expectation value of OAM at each atomic site, showing that 〈*L*_*x*_〉 = 〈*L*_*y*_〉 = 0 and 〈*L*_*z*_〉 is proportional to ${t}_{{p}_{x}{p}_{y}}\,\sin (ka)$. Thus, nonzero ${t}_{{p}_{x}{p}_{y}}$ is crucial for nonzero expectation value of OAM. Furthermore, 〈*L*_*z*_〉 is zero if the off-diagonal element of *H*_0_(*k*) is real. Thus, being imaginary of the *i* sin(*ka*) term, which originates from the change of the sign of the hopping energy depending on the hopping direction, is also crucial for nonzero expectation value of OAM.

Similarly, if an atomic chain has *d*_*xy*_ and ${d}_{{x}^{2}-{y}^{2}}$ orbitals at each atomic site [Fig. 1(c,d)], 〈*L*_*z*_〉 is proportional to the interatomic hopping energy between the *d*_*xy*_ and ${d}_{{x}^{2}-{y}^{2}}$ orbitals. Thus, the expectation value of OAM is zero if the chain has a mirror plane parallel to the *xz* plane [Fig. 1(c)], while it is nonzero if the chain has no mirror plane parallel to the *xz* plane [Fig. 1(d)]. To summarize, nonzero imaginary parts of the off-diagonal elements of *H*_0_(*k*) produce nonzero OAM, and it requires interatomic hopping between different-parity orbitals which is generated by specific broken mirror symmetry in the atomic structure.

In the literature, tight-binding models were developed previously to consider the spin-orbit interaction in various systems such as spin splitting in a square lattice of *s* orbitals connected by *p* orbitals^34^, the Rashba-type spin splitting of the Au (111) surface states^7^, and the spin splitting of *d* bands in perovskite transition-metal oxide structures^35–38^. In these studies, unquenched OAM might be obtained straightforwardly, but the OAM was not analyzed separately from SOI. Meanwhile, in the study of the Rashba-type spin splitting in ferroelectric halide perovskites^39^, OAM was obtained from the tight-binding model to show its chiral behavior, but structural and orbital conditions for nonzero OAM were still not analyzed.

Density functional theory (DFT) calculation is necessary to make a correct tight-binding model^40^, and our above tight-binding analysis for nonzero OAM can guide DFT-based analysis for understanding and control of OAM. Once important orbitals are recognized in DFT calculations, symmetry analysis can be made with a simple tight-binding model, leading to structural and orbiral conditions for unquenching of OAM. For example, a tight-binding model of *p* orbitals was previously conceived to explain GSS of the Au (111) surface states^7^, but the splitting was later found mainly due to spin-*d*-orbital interaction by first-principles calculations^28^. Thus, one needs a tight-binding model including *d* orbitals to analyze GSS of Au (111) surface states, as we will present in the next section.

In addition, it was found previously that GSS occurs together with spatial asymmetry of orbital and charge distribution^15, 17, 28^. In our present work, we note that it is the unquenched OAM that generates GSS while the spatially asymmetric distribution of the wavefunction is a result of mixing of different-parity orbitals.

### Density functional calculations

Now, we consider real materials such as WSe_2_ monolayer, Au (111) surface, and bulk HgTe. We also consider WSe_2_ monolayers perturbed by pressure, electric field, and substrate.

#### WSe_2_ mononlayer

**Figure 2:**
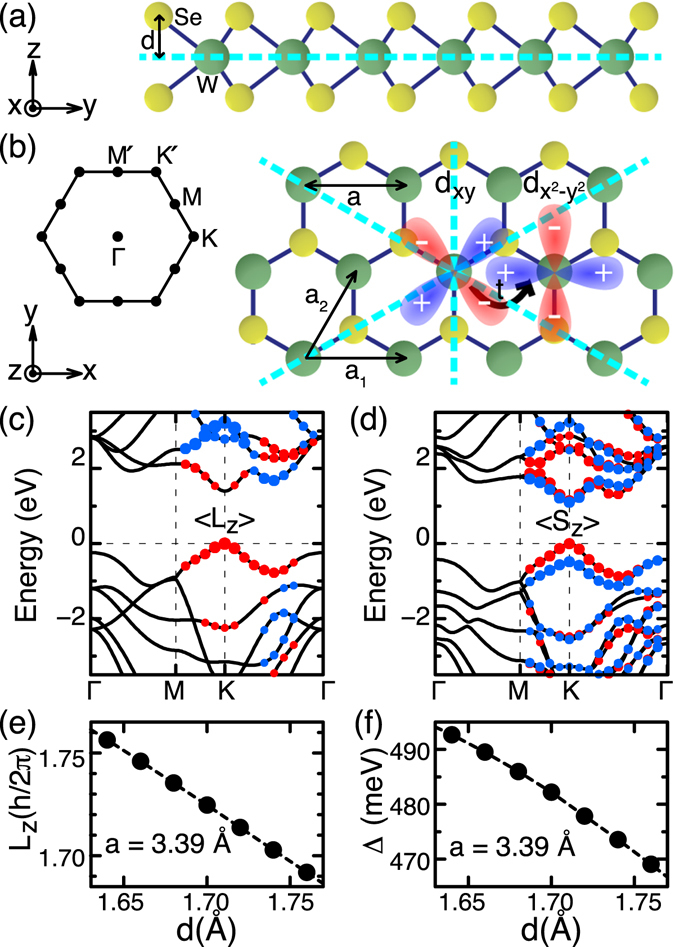
WSe_2_ monolayer. (**a**) Side and (**b**) top view. Dashed lines are mirror planes. The interatomic hopping between *d*_*xy*_ and ${d}_{{x}^{2}-{y}^{2}}$ orbitals of W atoms is nonzero in the *x* direction. The inset in (**b**) shows BZ, where the Γ-K direction is along the *x* axis. (**c**,**d**) Electronic bands obtained (**c**) without SOI and (**d**) with SOI. The size of dots represents |〈*L*_*z*_〉| obtained without SOI in (**c**) and |〈*S*_*z*_〉| with SOI in (**d**), with blue for positive and red for negative values. (**e**) The OAM |〈*L*_*z*_〉|, without SOI, and (**f**) the spin splitting Δ, with SOI, of the valence band maximum at K versus the distance *d* between W and Se planes. For the pristine monolayer, *d* is 1.7 Å.

As the first case of real materials, we consider the WSe_2_ monolayer, which shows GSS at valence bands near the K point in Brillouin zone (BZ)^41–43^. As shown in Fig. 2(a), the WSe_2_ monolayer has a mirror symmetry with respect to the *xy* plane containing W atoms. Consequently, 〈*L*_*x*_〉 and 〈*L*_*y*_〉 at W sites are always zero. Moreover, for a *k* vector along the Γ-M line, 〈*L*_*z*_〉 is also zero due to an additional mirror plane which is perpendicular to the *xy* plane and contains the *k* vector, as marked in dashed lines in Fig. 2(b). However, for a *k* vector off the Γ-M line, there is no such additional mirror symmetry, so 〈*L*_*z*_〉 may be nonzero. Our DFT calculation without SOI shows that 〈*L*_*z*_〉 of valence bands increases gradually as the *k* vector approaches the K point, where it becomes maximum, as shown in Fig. 2(c).

Our DFT calculation without SOI also shows that the highest valence band of the WSe_2_ monolayer consists mostly of *d*-orbitals of W atoms, consistent with previous reports^41, 42, 44^. In addition, the mirror symmetry with respect to the *xy* plane reduces the number of contributing *d* orbitals further^45^. Having even parity with respect to the *xy* plane, the valence band is composed of ${d}_{{z}^{2}}$, *d*_*xy*_, and ${d}_{{x}^{2}-{y}^{2}}$ orbitals only. As the ${d}_{{z}^{2}}$ orbital is an eigenstate with *L*_*z*_ = 0, only *d*_*xy*_ and ${d}_{{x}^{2}-{y}^{2}}$ orbitals can contribute to nonzero value of 〈*L*_*z*_〉. When we consider the valence-band states with *k* along the Γ-K line in the *k*_*x*_ direction, we find that 〈*L*_*z*_〉 is proportional to the interatomic hopping energy ${t}_{{d}_{xy}{d}_{{x}^{2}-{y}^{2}}}$ between *d*_*xy*_ and ${d}_{{x}^{2}-{y}^{2}}$ orbitals along the *x* direction, which is nonzero due to the presence of Se ions [Fig. 2(b)], similarly to the atomic chain in Fig. 1(d). Note that due to different parity of the two *d* orbitals with respect to the *yz* plane normal to the *k*_*x*_ direction, the sign of the hopping energy depends on the hopping direction, that is, the hopping energy from the *d*_*xy*_ to the ${d}_{{x}^{2}-{y}^{2}}$ orbital along the −*x* direction has opposite sign of that along the +*x* direction, producing imaginary off-diagonal elements of the Hamiltonian which is necessary for nonzero 〈*L*_*z*_〉. In summary, 〈*L*_*z*_〉 is nonzero along the Γ-K line in the WSe_2_ monolayer due to the interatomic hopping between *d* orbitals of different parities which is produced by specific mirror symmetry breaking by the presence of Se atoms.

In the WSe_2_ monolayer, nonzero 〈*L*_*z*_〉, which is proportional to ${t}_{{d}_{xy}{d}_{{x}^{2}-{y}^{2}}}$ as described above, results in a GSS in our DFT calculation with SOI [Fig. 2(d)] due to the strong atomic SOI of W atoms. Since SOI can be regarded effectively as ${H}_{SO}(\overrightarrow{k})={\lambda }_{5d}^{W}\overrightarrow{S}\cdot \overrightarrow{L}$, where ${\lambda }_{5d}^{W}$ is the atomic SOI strength of W 5*d* orbitals, the valence-band state ${\psi }_{\overrightarrow{K}}$ at the K point is spin-split by ${{\rm{\Delta }}}_{\overrightarrow{K}}=\hslash {\lambda }_{5d}^{W}\langle {\psi }_{\overrightarrow{K}}|{L}_{z}|{\psi }_{\overrightarrow{K}}\rangle$, with the spin aligned in either +*z* or −*z* direction. Here, $\langle {\psi }_{\overrightarrow{K}}|{L}_{z}|{\psi }_{\overrightarrow{K}}\rangle$ is the expectation value over a single W atom because the unit cell has only one W atom; otherwise, it should be sum over W atoms. Our approach thus shows straightforwardly that the spin is polarized completely out of plane and has three-fold rotational symmetry. This agrees with previous DFT calculations^41, 42^, and is consistent with anisotropic two-dimensional models^12, 30^ except that in-plane spin components present in the models are absent in DFT results.

When we consider a line of W atoms along Γ-K’ direction, Se atoms are on the right-hand side of the W-atom chain, as shown in Fig. 2(b). This is different from the case of the W-atom chain along Γ-K direction, where Se atoms are on the left-hand side of the W-atom chain, as shown in Fig. 2(b). This difference in the relative positions of Se atoms with respect to each W-atom chain makes the interorbital hopping energies along the two W-atom chains have opposite sign, resulting in opposite spin directions of the valence band maxima at K and K’, that is, the valley-dependent GSS in WSe_2_ monolayer^41–43^.

#### WSe_2_ monolayer under external perturbation

In this subsection, we consider WSe_2_ monolayers under uniaxial compression, in external electric field, and on top of a substrate. With these perturbations, we analyze the change in OAM without considering SOI, and then the change in the spin spliting with considering SOI.

As shown in the previous subsection, the interatomic hopping energy between *d*_*xy*_ and ${d}_{{x}^{2}-{y}^{2}}$ orbitals of W atoms is generated by the parity-breaking electric field from Se atoms, so the hopping energy will be increased if Se atoms move closer to W atoms. When the hopping energy is increased, 〈*L*_*z*_〉 will be increased and thereby the spin splitting will also be increased. We can demonstrate this by compressing the WSe_2_ monolayer along the *z* direction uniaxially. Our density functional calculations show that both the size of 〈*L*_*z*_〉, which is obtained without SOI, and the spin splitting, which is obtained with SOI, increase as the Se atoms are shifted toward the *xy* plane of W atoms by uniaxial compression [Fig. 2(e,f)].

**Figure 3:**
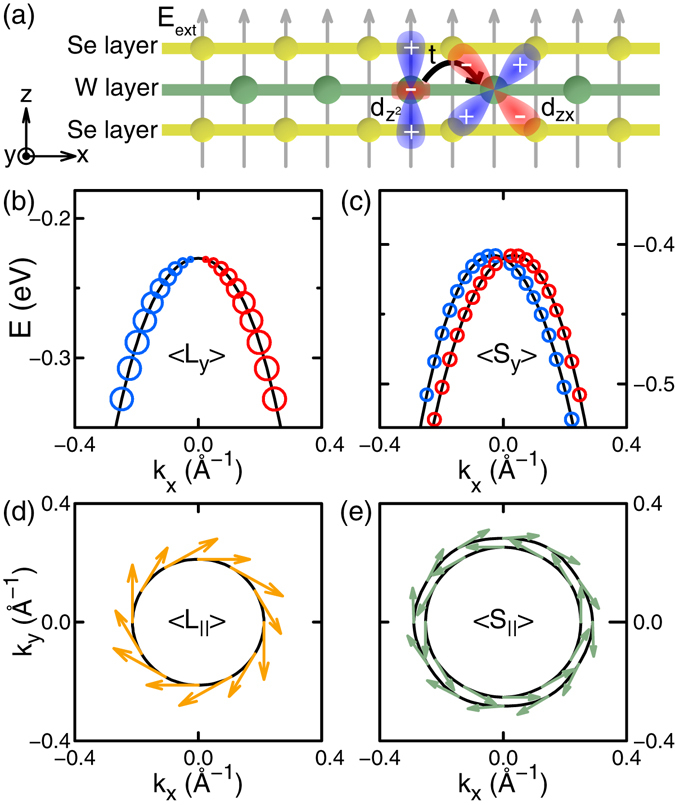
WSe_2_ monolayer under external electric field *E*_*ext*_ = 1 V/Å. (**a**) Schematic representation of the interatomic hopping between ${d}_{{z}^{2}}$ and *d*_*zx*_ orbitals of W atoms. (**b**,**c**) The highest valence band(s) near Γ obtained (**b**) without SOI and (**c**) with SOI, where 0 eV is the valence band maximum at K in each case. The size of open dots represents |〈*L*_*y*_〉| obtained without SOI in (**b**) and |〈*S*_*y*_〉| obtained with SOI in (**c**), with blue for positive and red for negative values. (**d**,**e**) Constant-energy cut of the highest valence band(s) near Γ obtained (**d**) without SOI at the energy of −0.3 eV and (**e**) with SOI at −0.5 eV. Arrows are in-plane components of OAM obtained without SOI in (**d**) and those of the spin obtained with SOI in (**e**). Although it is rather large to be applied, 1 V/Å is chosen for clear presentation. The spin splitting is found proportional to *E*_*ext*_ in the range from 0 to 2 V/Å.

Applying an external electric field *E*_*ext*_ is a well-known method for spin splitting^4, 5, 42, 46^. In our present work, we focus on OAM induced by *E*_*ext*_ before we consider GSS. We performed DFT calculations with *E*_*ext*_ normal to the WSe_2_ monolayer [Fig. 3(a)]. Since *E*_*ext*_ breaks the mirror symmetry with respect to the *xy* plane, the in-plane component, 〈*L*_*x*_〉 or 〈*L*_*y*_〉, of OAM can have a nonzero value. With the electric field, the highest valence band near Γ, consisting mainly of ${d}_{{z}^{2}}$ orbital, comes to have *d*_*yz*_ or *d*_*zx*_ orbital as well, which is not allowed without the field. Along the *k*_*x*_ (*k*_*y*_) direction, ${d}_{{z}^{2}}$ orbital is coupled with *d*_*zx*_ (*d*_*yz*_) orbital by the interatomic hopping between the orbitals [Fig. 3(a)] and this produces nonzero 〈*L*_*y*_〉 (〈*L*_*x*_〉). The expectation value of OAM without SOI and that of the spin with SOI are perpendicular to the *k*-vector, showing chiral behaviors around Γ, as shown in Fig. 3(b–e). The slight anisotropy in the band splitting in Fig. 3(e) is due to $\overrightarrow{k}$-dependent variation of 〈*L*_*z*_〉.

The presence of a substrate can produce similar effects to the case of the electric field. Our DFT calculations show that WSe_2_ monolayer on top of the Bi bilayer have chiral behaviors of OAM near the top of the valence bands at Γ, because the Bi bilayer plays a similar role to the external field *E*_*ext*_ in breaking the mirror symmetry. Resulting spin splitting near Γ is consistent with the previous report^47^.

#### Au (111) surface

**Figure 4:**
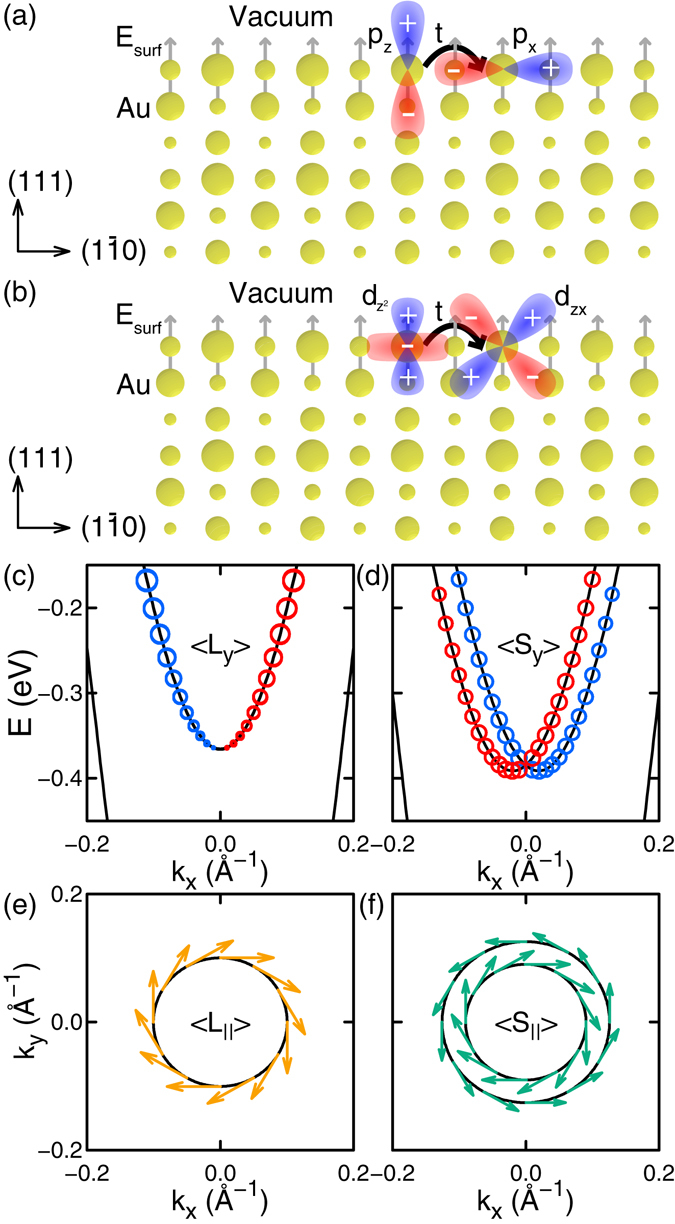
Au (111) surface. (**a**) Schematic representation of the interatomic hopping energy between *p*_*z*_ and *p*_*x*_ orbitals and (**b**) that between ${d}_{{z}^{2}}$ and *d*_*zx*_ orbitals of topmost surface Au atoms. The *x* axis is along $[1\bar{1}0]$ direction and the *z* axis is along [111] direction. (**c**) The spin-degenerate surface band near Γ obtained by DFT calculation without considering SOI. The size of open dots is proportional to |〈*L*_*y*_〉|. (**d**) The spin-split surface bands near Γ obtained with considering SOI. The size of open dots is proportional to |〈*S*_*y*_〉|. In (**c**,**d**), blue and red dots are for positive and negative values, respectively. (**e**,**f**) Constant-energy cut of the surface band(s) at −0.2 eV near Γ (**e**) without SOI and (**f**) with SOI. Arrows are in-plane components of OAM obtained without SOI in (**e**) and those of the spin with SOI in (**f**).

The well-known Rashba-type splitting of the Au (111) surface states^28, 48^ can be explained by the interorbital-hopping mechanism for nonzero OAM. Our DFT calculations show the Au (111) surface states consist of 6*s*, 6*p*, and 5*d* orbitals. At Γ, because of the symmetry of the system, the surface states consist of *s*, *p*_*z*_, and ${d}_{{z}^{2}}$ orbitals only, and OAM and the spin splitting are zero. Along the *k*_*x*_ (*k*_*y*_) direction near Γ, *p*_*x*_ (*p*_*y*_) and *d*_*zx*_ (*d*_*yz*_) orbitals also contribute to the surface states. At the Au (111) surface, the presence of the surface generates interatomic hopping between the *p*_*z*_ and the *p*_*x*_ (*p*_*y*_) orbital and that between the ${d}_{{z}^{2}}$ and the *d*_*zx*_ (*d*_*yz*_) orbital, as shown in Fig. 4(a,b), respectively. These interorbital hoppings produce nonzero 〈*L*_*y*_〉 (〈*L*_*x*_〉) which is linear in *k*_*x*_ (*k*_*y*_), resulting in chiral behaviors of OAM and the spin around Γ, as shown in Fig. 4(c–f). The interatomic hopping between *p* orbitals and that between *d* orbitals have opposite signs to each other so that generated *l* = 1 and *l* = 2 parts of OAM have opposite directions to each other. Meanwhile, the *p*-orbital contribution to the splitting is relatively small compared with the *d*-orbital one because the atomic SOI strength of the *p* orbitals is smaller than that of the *d* orbitals due to different radial extents of Au 6*p* and 5*d* orbitals. Thus, the 5*d* orbitals play a crucial role in the spin splitting despite the 6*s* and 6*p* orbitals are dominant orbitals of the surface states, consistently with the previous study^28^.

#### bulk HgTe

As shown below, interorbital-hopping mechanism for nonzero OAM is also applicable to Dresselhaus-type GSS in bulk systems which is described by an effective Hamiltonian of ${H}_{D}=\gamma \{{k}_{x}({k}_{y}^{2}-{k}_{z}^{2})\,{\sigma }_{x}+{k}_{y}({k}_{z}^{2}-{k}_{x}^{2})\,{\sigma }_{y}+{k}_{z}({k}_{x}^{2}-{k}_{y}^{2})\,{\sigma }_{z}\}$ for zinc blende structure^2, 3^.

**Figure 5:**
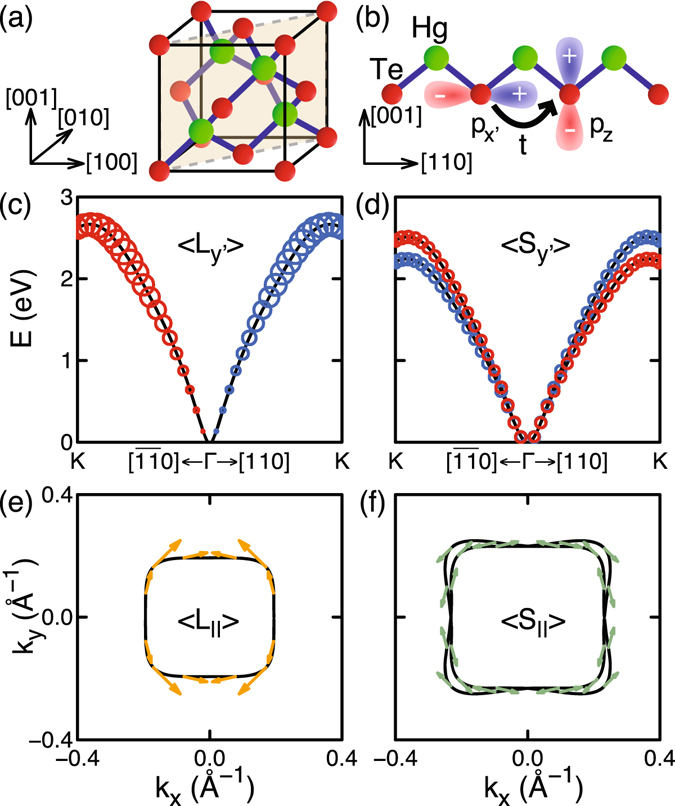
Bulk HgTe. (**a**) Atomic structure. The $(\bar{1}10)$ mirror plane is shaded in yellow. (**b**) Schematic representation of the interatomic hopping between Te *p*_*x*′_ and *p*_*z*_ orbitals in the [110] direction. (**c**,**d**) The lowest conduction band obtained (**c**) without SOI and (**d**) with SOI. The size of open dots represents |〈*L*_*y*′_〉| obtained without SOI in (**c**) and |〈*S*_*y*′_〉| with SOI in (**d**), with blue for positive and red for negative values. (**e**,**f**) Constant-energy cut of the lowest conduction band(s) at the energy of 1 eV near Γ obtained (**e**) without SOI and (**f**) with SOI. Arrows indicate $\langle \overrightarrow{L}\rangle$ obtained without SOI in (**e**) and $\langle \overrightarrow{S}\rangle$ with SOI in (**f**).

We consider GSS of the lowest conduction band in bulk HgTe, a mother compound for topological materials^49–51^. HgTe has the zinc blende structure as shown in Fig. 5(a). Our DFT calculations show that the lowest conduction band in bulk HgTe consists mainly of *p* orbitals of Te atoms. The OAM and the spin splitting in the lowest conduction band are zero for *k* along the [100] direction due to two mirror planes, (011) and $(01\bar{1})$, containing the [100] direction. In contrast, for *k* along [110] (say, *x*′ direction), the $(\bar{1}10)$ plane is the only mirror plane containing the [110] direction, and the (001) plane is not a mirror plane because of Hg atoms above Te atoms [Fig. 5(a)]. This broken mirror symmetry produces interatomic hopping between *p*_*x*′_ and *p*_*z*_ orbitals [Fig. 5(b)], resulting in nonzero 〈*L*_*y*′_〉 and nonzero spin splitting Δ which increase with *k*_*x*′_ [Fig. 5(c,d)]. Here, the *y*′ direction is $[\bar{1}10]$. Meanwhile, for *k* along $[\bar{1}10]$, Hg atoms are placed below Te atoms in the Hg-Te zigzag chain along $[\bar{1}10]$, which changes the sign of the hopping energy between *p*_*y*′_ and *p*_*z*_ orbitals and thereby reverses the OAM direction, resulting in the dependence of OAM on the $\overrightarrow{k}$ direction shown in Fig. 5(e). The OAMs in Fig. 5(e) are obtained with DFT calculations without SOI. With SOI, we have the spin splitting shown in Fig. 5(f), whose dependence on the $\overrightarrow{k}$ direction agrees with the Dresselhaus term that reduces to ${H}_{D}=\gamma {k}_{x}{k}_{y}({k}_{y}{\sigma }_{x}-{k}_{x}{\sigma }_{y})$ for *k*_*z*_ = 0.

Since it was reported that the valence bands near Γ in bulk HgTe are sensitive to the calculation methods^52^, we also performed DFT calculations with the local density approximation (LDA) and the modified Becke-Johnson semilocal exchange functional (MBJLDA)^53^ in order to check the validity of our calculations of OAM and spin splitting of the conduction band. As shown in Supplementary Fig. S1, the OAM and the spin splitting of the conduction band are not sensitive to calculation methods, which verifies our analysis of OAM and GSS of the conduction band of bulk HgTe described above.

### Temperature dependence

In our above analysis, OAM is determined mainly by the interatomic hopping energy. Since the hopping energy depends on atomic positions, OAM can be affected by thermal expansion of the lattice and vibration of atoms. In this subsection, we discuss the temperature dependence of OAM originating from temperature-dependent positions and motions of atoms.

First, we consider thermal expansion of the lattice. As the thermal expansion increases the interatomic distance, it decreases the spatial overlap of orbitals of neighboring atoms, thus weakening the interatomic hopping. Because OAM is propotional to the interatomic hopping, the primary effect of the thermal expansion is to reduce the size of OAM. Then the size of the spin splitting also decreases because the atomic SOI strength is insensitive to the lattice constant. Quantitative analysis can be achieved straightforwardly by first-principle calculations using atomic structures measured at different temperatures. It is very interesting that HgTe has negative thermal expansion at low temperature^54–56^. In that temperature range, the spin splitting in HgTe may increase with temperature because of decrease of lattice constants.

Next, we consider dependence of OAM on thermal vibration of atoms around their equilibrium positions. When the temperature rises, more phonons are excited and atomic vibrations become stronger. Electronic states are affected by phonons because the matrix elements of the electronic Hamiltonian are dependent on atomic positions. The Hamiltonian for the coupled electron-phonon system can be expressed as, up to the second order of atomic displacements^57^,

2 $$\begin{matrix}H & = & \,{\sum }_{n,{\bf{k}}}{\varepsilon }_{n,{\bf{k}}}\,{c}_{n,{\bf{k}}}^{\dagger }{c}_{n^{\prime} ,{\bf{k}}}+\,{\sum }_{{\bf{q}},\nu }\hslash {\omega }_{{\bf{q}},\nu }(\,{a}_{{\bf{q}},\nu }^{\dagger }{a}_{{\bf{q}},\nu }+\frac{1}{2})\\  &  & +\,{\sum }_{n,n^{\prime} ,{\bf{k}}}\,{\sum }_{{\bf{q}},\nu }\,{g}_{n,n^{\prime} ,{\bf{k}},{\bf{q}},\nu }^{\mathrm{(1)}}\,{c}_{n,{\bf{k}}+{\bf{q}}}^{\dagger }\,{c}_{n^{\prime} ,{\bf{k}}}({a}_{{\bf{q}},\nu }+\,{a}_{-{\bf{q}},\nu }^{\dagger })\\  &  & +\,{\sum }_{n,n^{\prime} ,{\bf{k}}}\,{\sum }_{{\bf{q}},\nu }\,\,{\sum }_{{\bf{q}}^{\prime} ,\nu ^{\prime} }\,{g}_{n,n^{\prime} ,{\bf{k}},{\bf{q}},{\bf{q}}^{\prime} ,\nu ,\nu ^{\prime} }^{\mathrm{(2)}}\,{c}_{n,{\bf{k}}+{\bf{q}}+{\bf{q}}^{\prime} }^{\dagger }\,{c}_{n^{\prime} ,{\bf{k}}}({a}_{{\bf{q}},\nu }+\,{a}_{-{\bf{q}},\nu }^{\dagger })({a}_{{\bf{q}}^{\prime} ,\nu ^{\prime} }+\,{a}_{-{\bf{q}}^{\prime} ,\nu ^{\prime} }^{\dagger })\mathrm{\ ,}\end{matrix}$$

where ${c}_{n,{\bf{k}}}^{\dagger }$ (*c*_*n*,**k**_) is the creation (annihilation) operator of an electron in the *n*th band with the wave vector **k** and the band energy *ε*_*n*,**k**_ and ${a}_{{\bf{q}}\nu }^{\dagger }$ (*a*_**q***ν*_) is the creation (annihilation) operator of a phonon in the *ν*th branch with the wave vector **q** and the angular frequency *ω*_**q**,*ν*_. The coupling constant ${g}_{n,n^{\prime} ,{\bf{k}},{\bf{q}},\nu }^{\mathrm{(1)}}$ (${g}_{n,n^{\prime} ,{\bf{k}},{\bf{q}},{\bf{q}}^{\prime} ,\nu ,\nu ^{\prime} }^{\mathrm{(2)}}$) is related with the first (second) derivatives of onsite and hopping energies of electrons with respect to atomic displacements. With the adabatic approximation that atoms are much slower than electrons, the creation operator ${\tilde{c}}_{n,{\bf{k}}}^{\dagger }$ of the perturbed electronic state up to the square of atomic displacements is

3 $$\begin{matrix}{\tilde{c}}_{n,{\bf{k}}}^{\dagger } & = & {c}_{n,{\bf{k}}}^{\dagger }+\sum _{n^{\prime} ,{\bf{q}},\nu }{A}_{n^{\prime} ,n,{\bf{k}},{\bf{q}},\nu }{c}_{n^{\prime} ,{\bf{k}}\,+\,{\bf{q}}}^{\dagger }({a}_{{\bf{q}},\nu }+{a}_{-{\bf{q}},\nu }^{\dagger })\\  &  & +\sum _{n^{\prime} ,{\bf{q}},{\bf{q}}^{\prime} ,\nu ,\nu ^{\prime} }{B}_{n^{\prime} ,n,{\bf{k}},{\bf{q}},{\bf{q}}^{\prime} ,\nu ,\nu ^{\prime} }{c}_{n^{\prime} ,{\bf{k}}\,+\,{\bf{q}}\,+\,{\bf{q}}^{\prime} }^{\dagger }({a}_{{\bf{q}},\nu }+{a}_{-{\bf{q}},\nu }^{\dagger })({a}_{{\bf{q}}^{\prime} ,\nu ^{\prime} }+{a}_{-{\bf{q}}^{\prime} ,\nu ^{\prime} }^{\dagger }),\end{matrix}$$

where ${A}_{n^{\prime} ,n,{\bf{k}},{\bf{q}},\nu }=\frac{{g}_{n^{\prime} ,n,{\bf{k}},{\bf{q}},\nu }^{\mathrm{(1)}}}{{\varepsilon }_{n,{\bf{k}}}-{\varepsilon }_{n^{\prime} ,{\bf{k}}+{\bf{q}}}}$ and ${B}_{n^{\prime} ,n,{\bf{k}},{\bf{q}},{\bf{q}}^{\prime} ,\nu ,\nu ^{\prime} }=\frac{{g}_{n^{\prime} ,n,{\bf{k}},{\bf{q}},{\bf{q}}^{\prime} ,\nu ,\nu ^{\prime} }^{\mathrm{(2)}}}{{\varepsilon }_{n,{\bf{k}}}-{\varepsilon }_{n^{\prime} ,{\bf{k}}+{\bf{q}}+{\bf{q}}^{\prime} }}+{\sum }_{n^{\prime\prime} }\frac{{g}_{n^{\prime} ,n^{\prime\prime} ,{\bf{k}}+{\bf{q}},{\bf{q}}^{\prime} ,\nu ^{\prime} }^{\mathrm{(1)}}\,{g}_{n^{\prime\prime} ,n,{\bf{k}},{\bf{q}},\nu }^{\mathrm{(1)}}}{({\varepsilon }_{n,{\bf{k}}}-{\varepsilon }_{n^{\prime} ,{\bf{k}}+{\bf{q}}+{\bf{q}}^{\prime} })({\varepsilon }_{n,{\bf{k}}}-{\varepsilon }_{n^{\prime\prime} ,{\bf{k}}+{\bf{q}}})}$. Here ${\tilde{c}}_{n,{\bf{k}}}^{\dagger }$ is not normalized yet. With the electronic state perturbed by phonons, the OAM of the state will fluctuate and the magnitude of the fluctation will grow with temperature. For the temperature dependence of the mean value of OAM, we consider the expectation value $\langle n,{\bf{k}}|{\overrightarrow{L}}_{i}|n,{\bf{k}}\rangle$ of the orbital angular momentum operator ${\overrightarrow{L}}_{i}$ at the *i*th atom using the perturbed electronc state, obtaining its phonon-number dependence as

4 $$\langle n,{\bf{k}}|{\overrightarrow{L}}_{i}|n,{\bf{k}}\rangle ={\langle n,{\bf{k}}|{\overrightarrow{L}}_{i}|n,{\bf{k}}\rangle }_{0}+\sum _{{\bf{q}},\nu }{\rm{\Delta }}{\overrightarrow{L}}_{i,n,{\bf{k}},{\bf{q}},\nu }({N}_{{\bf{q}},\nu }+\frac{1}{2}).$$

Here ${\langle n,{\bf{k}}|{\overrightarrow{L}}_{i}|n,{\bf{k}}\rangle }_{0}$ is the expectation value with respect to the unperturbed state, ${\rm{\Delta }}{\overrightarrow{L}}_{i,n,{\bf{k}},{\bf{q}},\nu }$ is the change of OAM per phonon given by

5 $$\begin{matrix}{\rm{\Delta }}{\overrightarrow{L}}_{i,n,{\bf{k}},{\bf{q}},\nu } & = & 4\,{\rm{Re}}[\sum _{n^{\prime} }\frac{{g}_{n^{\prime} ,n,{\bf{k}},{\bf{q}},-{\bf{q}},\nu ,\nu }^{\mathrm{(2)}}{\langle n,{\bf{k}}|{\overrightarrow{L}}_{i}|n^{\prime} ,{\bf{k}}\rangle }_{0}}{{\varepsilon }_{n,{\bf{k}}}-{\varepsilon }_{n^{\prime} ,{\bf{k}}}}\\  &  & +\sum _{n^{\prime} ,n^{\prime\prime} }\frac{{g}_{n^{\prime} ,n^{\prime\prime} ,{\bf{k}}+{\bf{q}},-{\bf{q}},\nu }^{\mathrm{(1)}}\,{g}_{n^{\prime\prime} ,n,{\bf{k}},{\bf{q}},\nu }^{\mathrm{(1)}}{\langle n,{\bf{k}}|{\overrightarrow{L}}_{i}|n^{\prime} ,{\bf{k}}\rangle }_{0}}{({\varepsilon }_{n,{\bf{k}}}-{\varepsilon }_{n^{\prime} ,{\bf{k}}})({\varepsilon }_{n,{\bf{k}}}-{\varepsilon }_{n^{\prime\prime} ,{\bf{k}}+{\bf{q}}})}]\\  &  & +2\sum _{n^{\prime} ,n^{\prime\prime} }\frac{{g}_{n^{\prime\prime} ,n,{\bf{k}},{\bf{q}},\nu }^{\mathrm{(1)}\ast }\,{g}_{n^{\prime} ,n,{\bf{k}},{\bf{q}},\nu }^{\mathrm{(1)}}{\langle n^{\prime\prime} ,{\bf{k}}+{\bf{q}}|{\overrightarrow{L}}_{i}|n^{\prime} ,{\bf{k}}+{\bf{q}}\rangle }_{0}}{({\varepsilon }_{n,{\bf{k}}}-{\varepsilon }_{n^{\prime\prime} ,{\bf{k}}+{\bf{q}}})({\varepsilon }_{n,{\bf{k}}}-{\varepsilon }_{n^{\prime} ,{\bf{k}}+{\bf{q}}})}\\  &  & -2\sum _{n^{\prime} }\frac{{|{g}_{n^{\prime} ,n,{\bf{k}},{\bf{q}},\nu }^{\mathrm{(1)}}|}^{2}{\langle n,{\bf{k}}|{\overrightarrow{L}}_{i}|n,{\bf{k}}\rangle }_{0}}{{({\varepsilon }_{n,{\bf{k}}}-{\varepsilon }_{n^{\prime} ,{\bf{k}}+{\bf{q}}})}^{2}},\end{matrix}$$

and *N*_**q**,*ν*_ is the phonon number at temperature *T* given by ${N}_{{\bf{q}},\nu }=\frac{1}{\exp (\hslash {\omega }_{{\bf{q}},\nu }/{k}_{B}T)-1}$. In Eq. (5), Re$[\cdots ]$ is the real part of a complex number, ${(\cdots )}^{\ast }$ is the complex conjugate, and the last term with ${\langle n,{\bf{k}}|{\overrightarrow{L}}_{i}|n,{\bf{k}}\rangle }_{0}$ came from the normalization of the perturbed state. Thus, using Eqs (4) and (5), one can find the phonon contribution to the temperature dependence of the mean value of OAM quantitatively from numerical calculation of the electron-phonon coupling matrix elements. In the qualitative sense, phonons can increase or decrease OAM as a function of temperature, thus strengthening or weakening the spin splitting in the energy bands. In another aspect, OAM has fluctuating part due to phonons and electron spins are coupled to OAM via atomic SOI. This results in thermal broadening of the spin splitting of the energy bands and dephasing of spins in the system.

## Conclusion

In conclusion, we investigated unquenched OAM of electronic bands in solids and nanostructures with GSS. We analyzed conditions for nonzero OAM in simple atomic chains and in real materials such as WSe_2_ monolayer, Au (111) surface, and bulk HgTe by performing DFT calculations. In all these cases, specific broken mirror symmetry generates interatomic hopping between different-parity orbitals, which generates wavefunctions with nonzero OAM near heavy element nucleus. This OAM couples with the spin via strong atomic SOI of heavy atoms, such as W, Au, and Te, resulting in GSS. We also demonstrated that control of OAM is possible by pressure, external electric field, and substrate, which leads to the control of GSS, and discussed the temperature dependence of OAM. Our results highlight the unquenched OAM as one of the key ingredients for GSS, providing a simplified picture for design and control of GSS materials.

## Methods

We perform first-principles calculations with the generalized gradient approximation^58^ to DFT, norm-conserving pseudopotentials^59^, and localized pseudoatomic orbitals for wavefunctions, as implemented in the SIESTA code^60^. SOI is incorporated within fully relativistic *j*-dependent pseudopotentials^61^ in the *l*-dependent fully-separable nonlocal form using additional Kleinman-Bylander-type projectors^62, 63^. The experimental lattice parameter^64^ is used for bulk HgTe.

## Electronic supplementary material

Supplementary Information: Orbital angular momentum analysis for giant spin splitting in solids and nanostructures
