# Publisher Correction: Orbital angular momentum analysis for giant spin splitting in solids and nanostructures

**DOI:** 10.1038/s41598-018-26489-z

**Published:** 2018-06-26

**Authors:** Sehoon Oh, Hyoung Joon Choi

**Affiliations:** Department of Physics and IPAP, Yonsei University, Seoul, 03722 Korea

Correction to: *Scientific Reports* 10.1038/s41598-017-02032-4, published online 17 May 2017

This Article contains a typographical error in Equation 2, where:

$$\begin{array}{ccc}H & = & \sum _{n,{\bf{k}}}{\varepsilon }_{n,{\bf{k}}}{c}_{n,{\bf{k}}}^{\dagger }{c}_{n^{\prime} ,{\bf{k}}}^{}+\sum _{{\bf{q}},{\rm{\nu }}}\hslash {\omega }_{{\bf{q}},{\rm{\nu }}}({a}_{{\bf{q}},{\rm{\nu }}}^{\dagger }{a}_{{\bf{q}},{\rm{\nu }}}^{}+\frac{1}{2})\\  &  & +\sum _{n,n^{\prime} ,{\bf{k}}}\sum _{{\bf{q}},{\rm{\nu }}}{g}_{n,n^{\prime} ,{\bf{k}},{\bf{q}},{\rm{\nu }}}^{(1)}{c}_{n,{\bf{k}}+{\bf{q}}}^{\dagger }{c}_{n^{\prime} ,{\bf{k}}}^{}({a}_{{\bf{q}},{\rm{\nu }}}+{a}_{-{\bf{q}},{\rm{\nu }}}^{\dagger })\\  &  & +\sum _{n,n^{\prime} ,{\bf{k}}}\sum _{{\bf{q}},{\rm{\nu }}}\sum _{{\bf{q}}^{\prime} ,{\rm{\nu }}^{\prime} }{g}_{n,n^{\prime} ,{\bf{k}},{\bf{q}},{\bf{q}}^{\prime} ,{\rm{\nu }},{\rm{\nu }}^{\prime} }^{(2)}{c}_{n,{\bf{k}}+{\bf{q}}+{\bf{q}}^{\prime} }^{\dagger }{c}_{n^{\prime} ,{\bf{k}}}^{}({a}_{{\bf{q}},{\rm{\nu }}}+{a}_{-{\bf{q}},{\rm{\nu }}}^{\dagger })({a}_{{\bf{q}}^{\prime} ,{\rm{\nu }}^{\prime} }+{a}_{-{\bf{q}}^{\prime} ,{\rm{\nu }}^{\prime} }^{\dagger })\end{array}$$

should read:

$$\begin{array}{ccc}H & = & \sum _{n,{\bf{k}}}{\varepsilon }_{n,{\bf{k}}}{c}_{n,{\bf{k}}}^{\dagger }{c}_{n,{\bf{k}}}^{}+\sum _{{\bf{q}},{\rm{\nu }}}\hslash {\omega }_{{\bf{q}},{\rm{\nu }}}({a}_{{\bf{q}},{\rm{\nu }}}^{\dagger }{a}_{{\bf{q}},{\rm{\nu }}}^{}+\frac{1}{2})\\  &  & +\sum _{n,n^{\prime} ,{\bf{k}}}\sum _{{\bf{q}},{\rm{\nu }}}{g}_{n,n^{\prime} ,{\bf{k}},{\bf{q}},{\rm{\nu }}}^{(1)}{c}_{n,{\bf{k}}+{\bf{q}}}^{\dagger }{c}_{n^{\prime} ,{\bf{k}}}^{}({a}_{{\bf{q}},{\rm{\nu }}}+{a}_{-{\bf{q}},{\rm{\nu }}}^{\dagger })\\  &  & +\sum _{n,n^{\prime} ,{\bf{k}}}\sum _{{\bf{q}},{\rm{\nu }}}\sum _{{\bf{q}}^{\prime} ,{\rm{\nu }}^{\prime} }{g}_{n,n^{\prime} ,{\bf{k}},{\bf{q}},{\bf{q}}^{\prime} ,{\rm{\nu }},{\rm{\nu }}^{\prime} }^{(2)}{c}_{n,{\bf{k}}+{\bf{q}}+{\bf{q}}^{\prime} }^{\dagger }{c}_{n^{\prime} ,{\bf{k}}}^{}({a}_{{\bf{q}},{\rm{\nu }}}+{a}_{-{\bf{q}},{\rm{\nu }}}^{\dagger })({a}_{{\bf{q}}^{\prime} ,{\rm{\nu }}^{\prime} }+{a}_{-{\bf{q}}^{\prime} ,{\rm{\nu }}^{\prime} }^{\dagger })\end{array}$$

