# Supplementary material for: Orbital angular momentum analysis for giant spin splitting in solids and nanostructures
Source: Sci Rep. 2017 May 17;7:2024. doi: 10.1038/s41598-017-02032-4 (PMC5435738; doi:10.1038/s41598-017-02032-4)
Supplement: Supplementary file 1 — Supplementary Information: Orbital angular momentum analysis for giant spin splitting in solids and nanostructures [file 41598_2017_2032_MOESM1_ESM.pdf]

Supplementary Information: Orbital angular momentum analysis for giant spin splitting in solids and nanostructures

Sehoon Oh[1] and Hyoung Joon Choi[1,*]

[1]Department of Physics and IPAP, Yonsei University, Seoul 03722, Korea
[*]h.j.choi@yonsei.ac.kr

ABSTRACT

This supplementary information provides comparison of overall band structure, orbital angular momentum, and spin splitting of bulk HgTe obtained with various calculation methods.

Supplementary information for bulk HgTe

As shown in Supplementary Fig. S1, the valence bands of HgTe near Γ at the energy range of -1 to 0 eV depend sensitively on the atomic structure and the use of the hybrid functional for the exchange-correlation energy in the density functional theory (DFT). However, the spin-split conduction bands from Γ to K, which are our current interest, are not sensitive to these, showing the gradual increase of orbital angular momentum (OAM) from Γ to K along with the increase of the spin splitting, robustly.

The spin-split valence bands in Supplementary Fig. S1(a), which are obtained by considering the spin-orbit interaction (SOI) and the experimental lattice parameter [1], seem to cross with each other at Γ near the energy of -1 eV. This, however, is not an actual crossing but there is a tiny energy splitting which is accidentally very small. This tiny energy splitting becomes substantial when the atomic structure is relaxed within DFT without any change of the crystal symmetry, as shown in Supplementary Fig. S1(b). This feature of the valence bands does not depend on whether the generalized gradient approximation (GGA) or the local density approximation (LDA) is used, as shown in Supplementary Figs. S1(b) and (c), and neither on whether the SIESTA code or the VASP code is used, as shown in Supplementary Figs. S1(b)-(e).

Since the SIESTA code does not have the modified Becke-Johnson semilocal exchange functional (MBJLDA) [2], the VASP code is used to calculate MBJLDA band structures [Supplementary Fig. S1(f)], and the OAM and the spin angular momentum (SAM) are obtained from the calculated conduction-band wave functions.

Our MBJLDA band structure [Supplementary Fig. S1(f)] agrees well with Fig. 1(b) of Ref. [3], verifying our calculational results. The OAM, the SAM, and the spin splitting in Supplementary Fig. S1(f) agree well with those in Supplementary Fig. S1(a). This shows that these conduction-band properties of HgTe do not depend sensitively on the detailed atomic structures and the types of the exchange-correlation functional.

[1] Wyckoff, R. W. G. Crystal Structures, (2nd ed.) vol. 1, p. 110. (Interscience, New York, 1960).
[2] Tran, F. & Blaha, P. Accurate band gaps of semiconductors and insulators with a semilocal exchange-correlation potential. *Phys. Rev. Lett.* **102**, 226401 (2009).
[3] Küfner, S. & Bechstedt, F. Topological states in α-Sn and HgTe quantum wells: a comparison of *ab initio* results. *Phys. Rev. B* **91**, 035311 (2015).

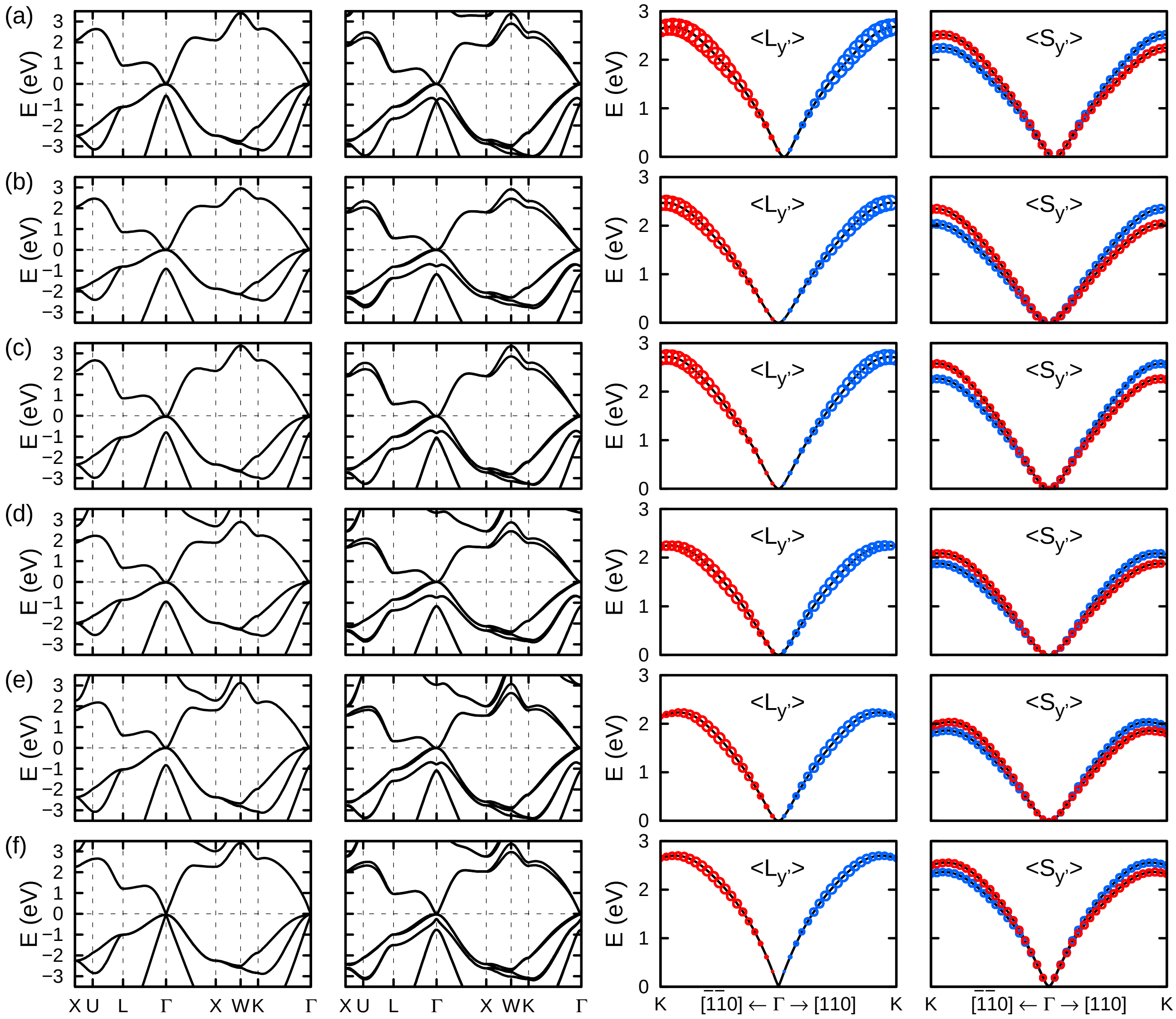

Figure S1. Electronic band structures, OAM, and SAM of bulk HgTe obtained by using (a) the SIESTA code with GGA and the experimental lattice parameter [1], (b) the SIESTA code with GGA and the fully relaxed atomic structure, (c) the SIESTA code with LDA and the fully relaxed atomic structure, (d) the VASP code with GGA and the fully relaxed atomic structure, (e) the VASP code with LDA and the fully relaxed atomic structure, and (f) the VASP code with MBJLDA [2] and the fully relaxed atomic structure with LDA. Each row shows, from left to right, (i) the band structure obtained without SOI, (ii) the spin-split band structure obtained with SOI, (iii) the lowest conduction band and their OAM obtained without SOI, where the radius of open dots is proportional to $|\langle L_{y'} \rangle|$, with blue for positive and red for negative values, and (iv) the spin-split lowest conduction bands and their SAM obtained with SOI, where the radius of dots is proportional to $|\langle S_{y'} \rangle|$, with blue for positive and red for negative values. Here the y' direction is [$\bar{1}10$].
